# Aging augments the impact of influenza respiratory tract infection on mobility impairments, muscle-localized inflammation, and muscle atrophy

**DOI:** 10.18632/aging.100882

**Published:** 2016-02-07

**Authors:** Jenna M. Bartley, Sarah J. Pan, Spencer R. Keilich, Jacob W. Hopkins, Iman M. Al-Naggar, George A. Kuchel, Laura Haynes

**Affiliations:** ^1^ UConn Center on Aging, University of Connecticut Health Center, Farmington, CT 06030, USA; ^2^ Department of Immunology, University of Connecticut Health Center, Farmington, CT 06030, USA

**Keywords:** influenza, muscle atrophy, aging, disability, resilience

## Abstract

Although the influenza virus only infects the respiratory system, myalgias are commonly experienced during infection. In addition to a greater risk of hospitalization and death, older adults are more likely to develop disability following influenza infection; however, this relationship is understudied. We hypothesized that upon challenge with influenza, aging would be associated with functional impairments, as well as upregulation of skeletal muscle inflammatory and atrophy genes. Infected young and aged mice demonstrated decreased mobility and altered gait kinetics. These declines were more prominent in hind limbs and in aged mice. Skeletal muscle expression of genes involved in inflammation, as well as muscle atrophy and proteolysis, increased during influenza infection with an elevated and prolonged peak in aged mice. Infection also decreased expression of positive regulators of muscle mass and myogenesis components to a greater degree in aged mice. Gene expression correlated to influenza-induced body mass loss, although evidence did not support direct muscle infection. Overall, influenza leads to mobility impairments with induction of inflammatory and muscle degradation genes and downregulation of positive regulators of muscle. These effects are augmented and prolonged with aging, providing a molecular link between influenza infection, decreased resilience and increased risk of disability in the elderly.

## INTRODUCTION

It is well established that immune function declines with aging. Immunosenescence of both the innate and adaptive immune systems results in increased susceptibility to infection, as well as increased severity of infection in the elderly. Influenza (flu) tends to be particularly problematic in the elderly with increased risk for serious complications and hospitalization. Approximately 90% of flu-related deaths occur in the elderly [1], with influenza and pneumonia being the seventh leading cause of death among persons over 65 years old in the United States [2]. Even when death is avoided, elderly have increased risk of morbidity and disability from flu infection. Flu-related hospitalizations are associated with increased loss of independence [3] and long term declines in activities of daily living are observed post flu infection among nursing home residents [4]. Further, flu is among the leading causes of catastrophic disability and dramatic losses of activities of daily living in the elderly [5]. While it is known that prolonged hospitalization of the elderly is associated with decreased muscle mass and strength; flu infections, independent of hospitalization, have some degree of muscle involvement with myalgia among the common symptoms even in uncomplicated infections [6].

While myalgia is a common non-pulmonary symptom of flu infection, other myopathies are less commonly reported. Interestingly, despite increased clinical severity in the elderly, most flu associated myopathies are reported in pediatrics [7, 8], though it is possible, and quite likely, that elderly myopathies are under reported and not the primary focus of care due to other more life-threatening complications. In pediatric populations the most common flu-associated myopathy is acute myositis, characterized by severe calf pain, difficulty walking, and altered gait that generally resolves on its own within 30 days, but more commonly within a week [7, 8]. Less frequently acute myositis has also been reported in both adults [9] and the elderly [10]. In a range of ages, elevated circulating markers of muscle damage, such as creatine kinase (CK), myoglobin, and lactate dehydrogenase, have been reported during flu infection [7-13]. Additionally, during flu pandemics there have been cases of rhabdomyolysis reported [14-16] and muscle biopsies have confirmed atrophic/necrotic muscle fibers, though inflammatory cell infiltration seems less common [7, 17]. Furthermore, during the 2009 H1N1 flu pandemic, elevated serum CK was associated with worse flu outcomes (length of intensive care unit stay, increased pulmonary, kidney, and other non-pulmonary complications) [11].

Nevertheless, the flu virus demonstrates great specificity for pulmonary epithelial cells, with all evidence indicating that in all or nearly all cases active infection remains limited to the respiratory system [6]. Thus, while a wealth of literature indicates symptomatic or functional muscle involvement with pulmonary flu infection, it is unclear if these complications only occur in severe infections or if they are under reported and under studied in less severe infections. The limited research regarding flu-induced myopathy pathogenesis is controversial; direct viral infection of the muscle and immune-mediated cytokine storm induced muscle damage are among the top hypotheses. While some *in vitro* studies have shown that myoblasts and myotubes may be susceptible to infection and might produce live viral progeny [18-20], isolation of virus from muscle biopsies is rare [12, 21-23]. *In vivo* murine experiments showed that a non-permissive infection is possible in mature muscle fibers, though this is more likely with intramuscular inoculation [24-26], so the clinical relevance of these experiments remains entirely unclear.

While the pathogenesis of flu-associated myalgia and myopathy has yet to be determined, their clinical significance is apparent. Though flu-induced myopathies in pediatric cases are not long lasting conditions with permanent effects, it is possible that due to decreased resilience in the elderly flu-induced myopathies may be prolonged and have lasting effects; leading to the increased disability and loss of independence observed post flu [3-5, 27]. As the aging population continues to grow, emphasis on extending healthspan and increasing resilience is necessary [28]. Flu and possibly other respiratory tract infections may represent an under reported risk factor predisposing elderly to sarcopenia, frailty, and overall decreased resilience. Here, in a well-validated murine model of flu infection, we aimed to characterize flu effects on skeletal muscle, both from a functional and molecular perspective, in both young and aged mice. We hypothesized that during flu infection aging would be associated with diminished mobility and functional performance together with upregulation of skeletal muscle inflammatory and atrophy genes.

## RESULTS

### Following influenza infection aged mice have prolonged weight loss and elevated lung viral titers

**Figure 1:**
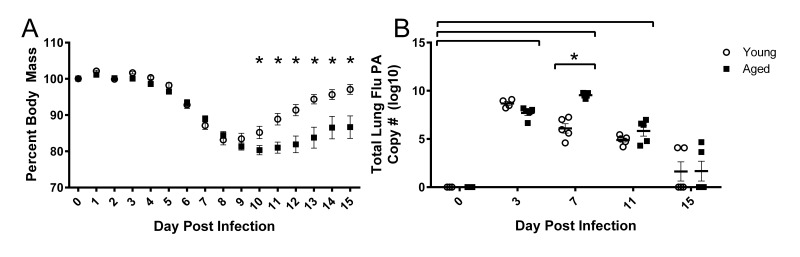
Prolonged weight loss and elevated lung viral titers in aged mice during influenza infection Young and aged C57BL/6 mice were intranasally infected with 500 EID_50_ of PR8 influenza. (**A**) Weight loss was monitored throughout the infection and percent weight loss was calculated from day 0 prior to infection. Significant weight loss (compared to day 0) was observed day 6 through 15 (not indicated in figure) and differences between young and aged mice were observed at time points indicated (* = p<0.05). Data shown as mean ± SEM and analyzed via two-way ANOVA with Bonferroni post hoc corrections. (**B**) On day 0, 3, 7, 11, and 15 whole lung tissue was harvested and RNA was isolated. Total influenza PA copy number was determined via RT-qPCR. Significant viral burden was observed following flu infection (compared to day 0, p<0.05, indicated by brackets above data) and differences between young and aged mice at time points indicated (* = p<0.05). Data analyzed via two-way ANOVA with Bonferroni post hoc corrections. Data shown from one independent experiment with individual samples as dots, mean and SEM indicated by line and error bars, respectively.

Young and aged mice were infected intranasally with a sublethal dose (500 EID_50_) of influenza A/PR/8/34 (PR8). Percent weight loss following infection is more severe and prolonged in aged mice (Fig 1A). While young mice begin to gain body mass by 10 days post infection (DPI), aged mice do not recover as quickly and differences between young and aged mice exist on 10-15 DPI. In the aged groups, increased weight loss is accompanied by slower viral clearance (Fig 1B) as measured by influenza polymerase (PA) copies in whole lung tissue via RT-qPCR. Although weight loss is a common marker used for pathogenicity in mice, it is rarely considered as a relevant outcome measure and mechanisms involved remain unknown.

### Influenza infection induces impairments in voluntary locomotor activity and gait parameters in young and aged mice

**Figure 2:**
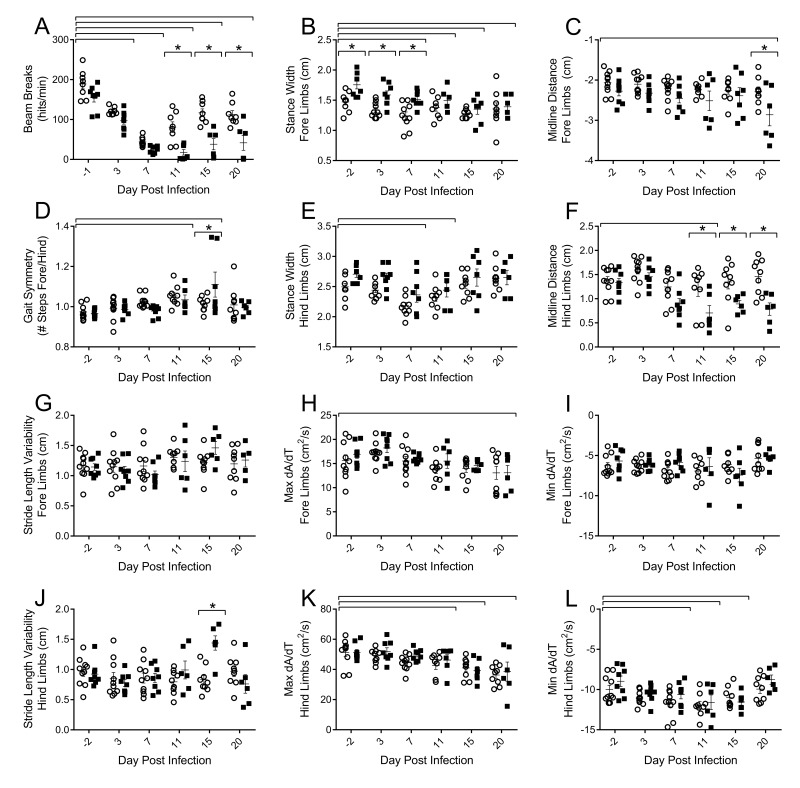
Influenza infection induced functional decrements in voluntary locomotor activity and gait kinematics that is more pronounced in the hind limbs and in the aged mice Young and aged C57BL/6 mice were intranasally infected with 500 EID_50_ of PR8 influenza. On days 0, 3, 7, 11, 15, and 20 mice were tested for functional performance. (**A**) Spontaneous voluntary activity was assessed via the open field test on a photobeam activity system. Beam breaks were recorded as mice traveled at 16”x16” open field and locomotor activity was assessed as beam breaks per minute. Gait parameters were assessed utilizing DigiGait, a ventral plane videography treadmill system. Postural gait parameters (Stance width of the fore (**B**) and hind (**E**) limbs and midline distance of the fore (**C**) and hind (**F**) limbs) were altered during flu infection with more prominent differences in the hind limbs of aged mice. Kinematic gait parameters were also altered with flu infection. Gait symmetry of the fore/hind limbs (**D**) was increased. Stride length variability of the fore limb (**G**) did not change, however the aged mice had increased stride length variability in the hind limbs later in the infection (**J**). Maximal rate of change of paw area contact during the breaking phase (Max dA/dt) and propulsion phase (Min dA/dt) is altered in the fore (**H** and **I**, respectively) and hind limbs (**K** and **L**, respectively) with more dramatic results in the hind limbs. All data analyzed via two-way ANOVA with Bonferroni post hoc corrections with effect of flu infection over time (compared to day 0, p<0.05) indicated by brackets above data and differences between young and aged mice (p<0.05) at time points indicated by asterisk.

To assess functional decrements associated with flu infection, we examined both voluntary activity levels, as well as more sensitive postural and kinematic gait alterations. Decreased voluntary locomotor activity was evident by 3 DPI in both young and aged mice as assessed by beam breaks in the open field test (Fig 2A). Diminished activity persisted through 20 DPI. Aged mice had fewer beam breaks per minute than their young counterparts on 11 (77.9% fewer), 15 (67.5% fewer), and 20 DPI (63.1% fewer) indicating that flu-induced decreased voluntary locomotor activity is more pronounced and prolonged in aged mice.

More detailed analysis of walking patterns was performed using the DigiGait system, which employs ventral plane videography to assess both spatial and temporal indices of gait at a given speed. Preliminary studies determined that 16cm/s was a speed that both young and aged mice could complete without difficulty and have consistent gait patterns for analysis (data not shown). Throughout the course of flu infection, no significant changes in stride, swing, or stance duration were observed (data not shown); however, alterations in postural components and acceleration parameters existed. By 7 DPI mice reduced stance width of both the fore (Fig 2B) and hind (Fig 2E) limbs by 15% and 13%, respectively, compared to baseline. Interestingly, aged mice initially had a wider fore limb stance, but these differences were concealed later in the infection. More pronounced differences in flu-induced gait alterations between the young and aged mice were observed in the midline distance of the fore (Fig 2C) and hind limbs (Fig 2F) later in infection, with hind limb midline distance being 46% narrower in aged mice compared to young mice on 20 DPI. This indicates aged mice are reaching less from their center with every step, perhaps due to increased muscle and joint pain limiting mobility. Additionally, later in the infection aged mice have increased stride length variability in the hind limbs compared to the young mice (Fig 2J). Gait symmetry index of the fore/hind limbs (Fig 2D) was increased in both young and aged mice at 11 DPI and 15 DPI as well, indicating a greater number of steps were taken with the fore limbs compared to the hind limbs.

Acceleration and deceleration measures were also affected by flu infection. The maximal rate of change of paw area contact during the braking phase (Max dA/dt), or how rapidly the mouse decelerates, is decreased in the fore (Fig 2H) and hind limbs (Fig 2K) later in the infection, though more pronounced in the hind limbs. Similarly, the maximal rate of change in paw area contact during the propulsion phase (Min dA/dt), or how rapidly the mouse propels itself into the next step, is decreased in the hind limbs by 7 DPI, but recovered by 20 DPI (Fig 2L). Both acceleration and deceleration are important parameters indicating the rate of force development, an important component of muscle health and quality. Interestingly, decreased Max dA/dt was still evident at 20 DPI.

Taken together, alterations in gait include decreased force development accompanied by decreased reaching distance and a narrower stance, more marked in the hind limbs and in aged mice. Diminished voluntary locomotor activity is prolonged in the aged mice as well. Functional impairments may be indicative of flu-induced muscle inflammation and damage, and that this is more severe and prolonged in aged mice.

### Influenza infection induces altered inflammatory gene expression in skeletal muscle

**Figure 3:**
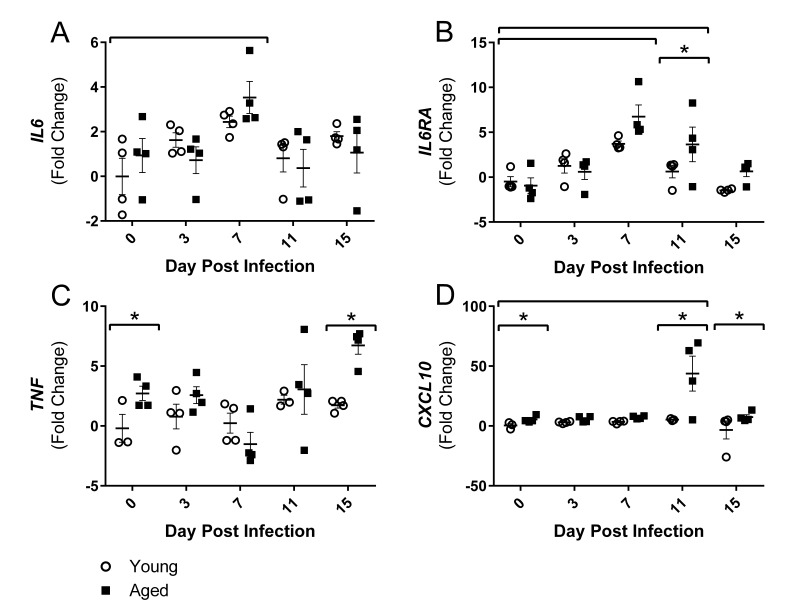
Influenza infection induced muscle-localized inflammatory gene expression in the gastrocnemius that is prolonged and elevated in aged mice Young and aged C57BL/6 mice were intranasally infected with 500 EID_50_ of PR8 influenza. At day 0, 3, 7, 11, and 15, mice fasted for 4-6 hours prior to sacrifice and gastrocnemius muscle was harvested and RNA was isolated. Gene expression was analyzed via RT-qPCR and normalized to reference genes and expression of young mice at day 0 to indicate fold changes. Influenza induced increased expression of *IL6* (**A**) and *IL6RA* (**B**). Increased *TNF* (**C**) and *CXCL10* (**D**) expression was observed in the aged mice. All data was log-transformed and analyzed via two-way ANOVA with Bonferroni post hoc corrections with effect of flu infection over time (compared to day 0, p<0.05) indicated by brackets above data and differences between young and aged mice (p<0.05) at time points indicated by asterisk.

Since alterations in gait were primarily in hind limbs, the gastrocnemius (gastroc) muscle gene expression was further examined over the course of flu infection in young and aged mice. As hypothesized, inflammatory gene expression in the gastroc was altered over the course of the flu infection. By 7 DPI flu induced increased gene expression of interleukin (IL)-6 (*IL6*) and IL-6 receptor alpha (*IL6RA*) (Fig 3A and Fig 3B).

*IL6RA* expression remained elevated by 3.6 fold in the aged mice at 11 DPI while the young mice returned to baseline expression levels. Aged gastroc had 2.7 fold increased expression of tumor necrosis factor (*TNF*) at baseline and these differences were intensified on 15 DPI with 6.7 fold greater expression of *TNF* in aged gastroc (Fig 2C). IL-6 and TNF are two key inflammatory mediators in skeletal muscle degeneration and repair, signaling through STAT3 and NFκB, respectively, that have vast effects on pro-inflammatory signaling, protein degradation, and atrophy gene induction (reviewed in [29]). Additionally, a dramatic 43 fold increase in expression of chemokine (C-X-C Motif) Ligand 10 (*CXCL10)*, a predominant player in T helper (Th) 1 responses that recruits immune cells, particularly T lymphocytes expressing its receptor CXCR3, into tissue, was observed in the aged gastroc at 11 DPI (Fig 3D). Prolonged and exaggerated levels of CXCL10 would increase immune cell recruitment and muscle inflammation, and potentially impair muscle regeneration processes.

Taking these results collectively, flu induced lingering inflammation in the aged muscle. As it is known that these inflammatory mediators signal through NFκB and other pathways that induce muscle atrophy and protein degradation, we next examined the expression of genes involved in these processes.

### Increased expression of protein degradation and muscle atrophy genes post influenza infection is prolonged in aged mice

**Figure 4:**
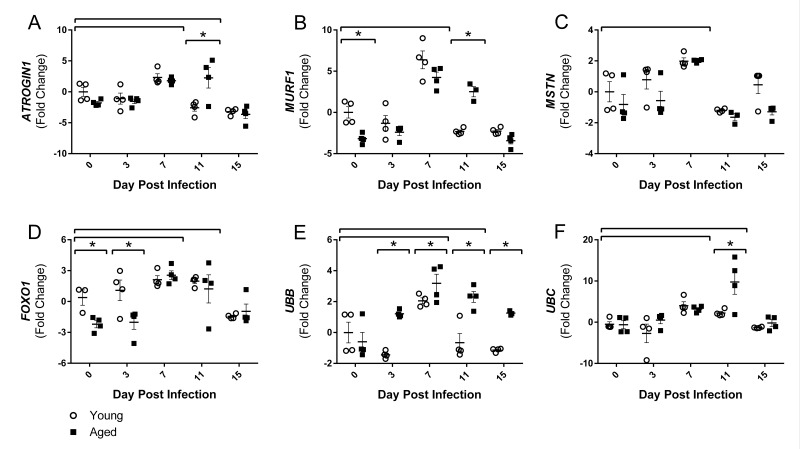
Influenza infection induced gastrocnemius expression ubiquitin proteasome pathway components that is more dramatic in aged mice Young and aged C57BL/6 mice were intranasally infected with 500 EID_50_ of PR8 influenza. At day 0, 3, 7, 11, and 15, mice were fasted for 4-6 hours prior to sacrifice and gastrocnemius muscle was harvested and RNA was isolated. Gene expression was analyzed via RT-qPCR and normalized to reference genes and expression of young mice at day 0 to indicate fold changes. Influenza induced increased skeletal muscle expression of negative muscle regulators (Myostatin (*MSTN*, **C**) and Forkhead box protein O1 (*FOXO1*, **D**), as well as ubiquitin proteasome components (*Atrogin1* (**A**), *MuRF1* (**B**), Ubiquitin B (*UBB*, **E**), and Ubiquitin C (*UBC*, **F**). All data was log-transformed and analyzed via two-way ANOVA with Bonferroni post hoc corrections with effect of flu infection over time (compared to day 0, p<0.05) indicated by brackets above data and differences between young and aged mice (p<0.05) at time points indicated by asterisk.

Although there are many pathways involved in protein degradation and atrophy, we focused on the ubiquitin proteasome pathway, primarily the muscle-specific E3 ubiquitin ligases atrogin-1 (also known as muscle atrophy F-box (MAFbx)) and muscle RING finger 1 (MuRF1), as the majority of literature to date shows increased atrogin-1 and/or MuRF1 expression at some point during almost all conditions of muscle wasting and atrophy [30]. As part of the ubiquitin proteasome pathway, atrogin-1 and MuRF1 control the ubiquitination and therefore degradation of specific target proteins in response to key signals. Key initiators of these pathways include inflammatory cytokines, as well as myostatin, glucocorticoids, FoxO transcription factors, and others. Following flu infection, we observed increased gastroc gene expression of both myostatin (*MSTN*) and *FOXO1* at 7 DPI (Fig 4C and 4D), by 2.0 and 2.3 fold change, respectively. *FOXO1* remained elevated at 11 DPI, while *MSTN* elevation was only transient. Interestingly, *FOXO1* was elevated in the young compared to aged mice at baseline and 3 DPI.

Corresponding with elevated cytokines, myostatin, and FOXO1 gene expression, both *ATROGIN1* and *MuRF1* gene expression were elevated with flu infection at 7 DPI by 2.1 and 5.3 fold, respectively. Moreover, in accordance with the prolonged inflammation, aged mice *ATROGIN1* and *MuRF1* expression remained elevated at 11 DPI (Fig 4A and Fig 4B). While in young mice *ATROGIN1 and MuRF1* expression was already decreased to below baseline levels (−2.6 and −2.3 fold, respectively) at this time point, aged mice expression remained elevated 2.3 and 2.5 fold, respectively. Interestingly, *ATROGIN1* was downregulated by 15 DPI, perhaps indicating an attempt to limit muscle degradation and begin repair processes. Baseline expression of *MuRF1* was decreased in aged mice, while no significant baseline differences existed in *ATROGIN1* expression. The influence of atrogin-1 and MuRF1 mRNA and protein expression on solely age-related muscle loss has yet to be resolved; some murine studies agree with our findings and show baseline suppression [31], while others have shown increased expression [32], and human studies have shown no differences [33, 34]. Nonetheless, induction of atrogin-1 and MuRF1 in response to flu infection indicates increased ubiquitination and proteolysis within the muscle. Furthermore, expression of two ubiquitin proteasome encoding genes, ubiquitin B (*UBB*) and ubiquitin C (*UBC*), was also increased with flu infection with a 2.6 and 3.6 fold increase at 7 DPI (Fig 4E and Fig 4F). Similarly, these increases were more dramatic and prolonged in aged mice. In young mice *UBC* expression peaked at 7 DPI, while aged mice continued increasing expression to a staggering 9.8 fold increase at 11 DPI. The increased levels of *UBB* and *UBC*, as well as *ATROGIN1* and *MuRF-1* indicate a catabolic environment of increased proteolysis, promoting muscle atrophy.

### Influenza infection decreases expression of positive regulators of muscle growth

**Figure 5:**
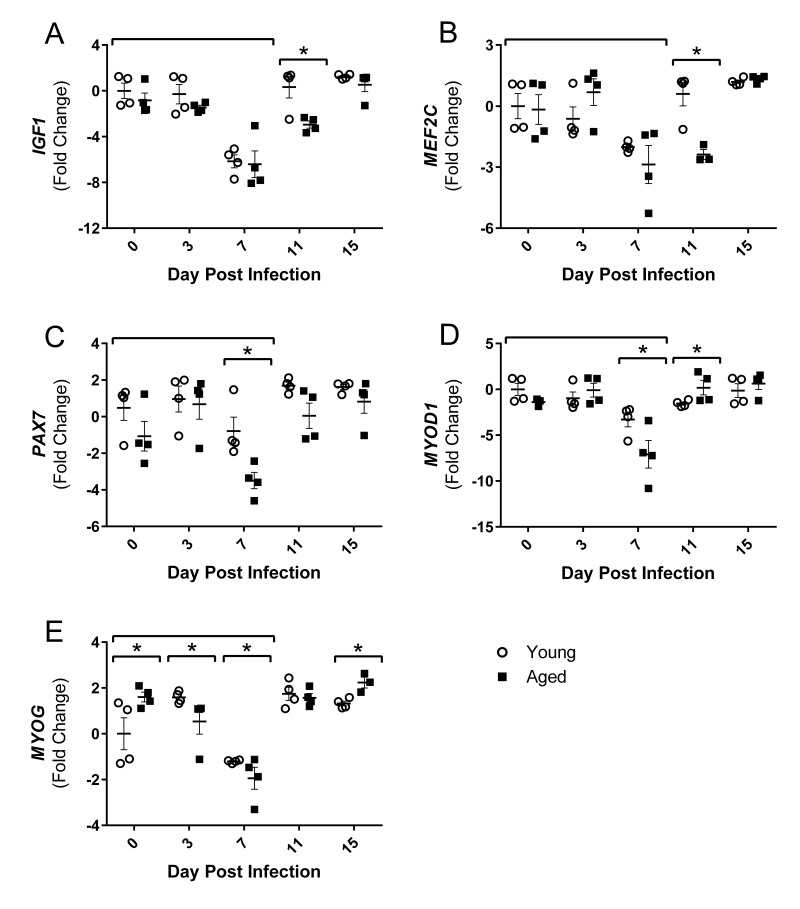
Influenza infection reduced gastrocnemius expression of positive regulators of muscle mass and myogenic regulatory factors to a greater degree in aged mice Young and aged C57BL/6 mice were intranasally infected with 500 EID_50_ of PR8 influenza. At day 0, 3, 7, 11, and 15, mice were fasted for 4-6 hours prior to sacrifice and gastrocnemius muscle was harvested and RNA was isolated. Gene expression was analyzed via RT-qPCR and normalized to reference genes and expression of young mice at day 0 to indicate fold changes. Influenza reduced skeletal muscle expression of insulin-like growth factor 1 (*IGF1*, **A**), myocyte enhancer binding factor 2C (*MEF2C*, **B**), paired box protein 7 (*PAX7*, **C**), myogenic differentiation 1 (*MYOD1*, **D**), and myogenin (*MYOG*, **E**). All data was log-transformed and analyzed via two-way ANOVA with Bonferroni post hoc corrections with effect of flu infection over time (compared to day 0, p<0.05) indicated by brackets above data and differences between young and aged mice (p<0.05) at time points indicated by asterisk.

Increased atrogin1/MuRF-1 pathway components, as well as increased inflammatory cytokines, are associated with diminished muscle growth; however, following injury or atrophy muscle generally has a remarkable capacity to regenerate and repair. Muscle regeneration through myogenesis is regulated at many key steps by myogenic regulatory factors (MRFs), myocyte enhancer binding factors 2 (Mef2), and other growth factors. Thus, we next examined the gene expression of positive regulators of muscle growth following flu infection to determine if repair processes are suppressed during this time and/or if muscle regeneration follows flu-induced atrophic responses. At 7 DPI when atrophy and ubiquitin genes are upregulated, gastroc *IGF1* expression is reduced in both young and aged mice (Fig 5A). This was transient in the young mice, but remained downregulated in the aged mice through 11 DPI. During flu infection gastroc *PAX7*, a marker of satellite cells which are critical for adding myonuclei and regenerating muscle tissue, expression is decreased at 7 DPI in both young and aged mice, but to a greater degree in aged mice, less than one fold reduction compared to ∼3.5 fold reduction in the aged (Fig 5D). Similarly at this time the expression of both *MYOD1* and *MYOG*, key MRFs, was decreased to a greater degree, with approximately 7 and 2 fold reductions, respectively, in the aged mice (Fig 5E and 5F). *MEF2C*, which acts in concert with the MRFs to control DNA binding and transcriptional regulation, was also suppressed at this time point and remained suppressed at 11 DPI in the aged mice (Fig 5B).

In summary, flu suppressed positive regulators of muscle mass and regeneration with concurrent increases in negative regulators. Additionally, these responses were greater and/or prolonged in the aged mice.

### Influenza-induced weight loss is correlated with gastroc gene expression of ubiquitin proteasome components

**Figure 6:**
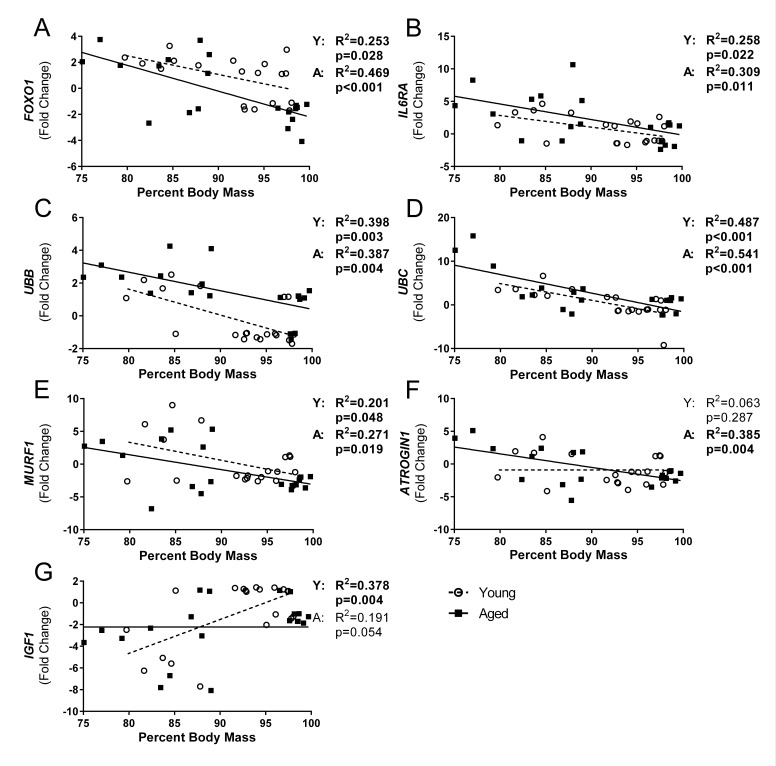
Influenza-induced weight loss correlated with gastrocnemius gene expression of ubiquitin proteasome pathway components Percent body mass loss at time of sacrifice and corresponding gastrocnemius gene expression was analyzed via univariate linear regression for all genes that showed significant time effects. Young and aged mice were analyzed separately to determine if relationships vary with age. *FOXO1* (**A**), *IL6RA* (**B**), *UBB* (**C**), *UBC* (**D**), *MURF1* (**E**), *ATROGIN1* (**F**), and *IGF1* (**G**) were significantly correlated with percent body mass in either young or aged, or both (Young (Y) and aged (A) mice regression analysis p and R^2^ values indicated to right of graph, bolded if significant (p<0.05)), while no relationship was seen with percent body mass and expression of *IL6*, *TNF*, *CXCL10*, *MEF2C*, *PAX7*, *MYOD1*, and *MYOG* (data not shown).

In order to determine if flu-induced weight loss was associated with muscle degradation, we performed correlation analysis on all variables that exhibited a time effect over the course of the flu infection. Indeed, multiple variables had significant correlations and age interactions were evident (Fig 6). In both young and aged mice *FOXO1* (Fig 6A), *IL6RA* (Fig 6B), *UBB* (Fig 6C), *UBC* (Fig 6D), and *MuRF1* (Fig 6E) were significantly correlated with percent body mass where *UBC* accounted for the greatest variation with R^2^ = 0.487 and 0.541 in young and aged mice, respectively (Fig 6D). Interestingly, a significant correlation was observed with *IGF1* expression in young mice that was not evident in aged mice (Fig 6G), and the opposite was observed with *ATROGIN1* expression (Fig 6F).

Further, all variables that had any significant correlations per age group were placed in a step-wise multiple regression analysis to determine if multiple variables could account for greater variability. In young mice *UBC, IGF1,* and *MuRF1* expression accounted for approximately 71% of variability seen in body mass changes (adjusted R^2^= 0.714, p=0.007), with additional variables not adding significantly to the model. In contrast, in aged mice *UBC* expression accounted for approximately 58% of the variability in body mass (adjusted R^2^= 0.576, p<0.001) with no other variables tested (*ATROGIN1, MuRF1, IL6RA, UBB*, and *FOXO1)* adding significantly to the model.

### Negligible viral copies detected in the gastrocnemius post influenza infection in vivo

**Figure 7:**
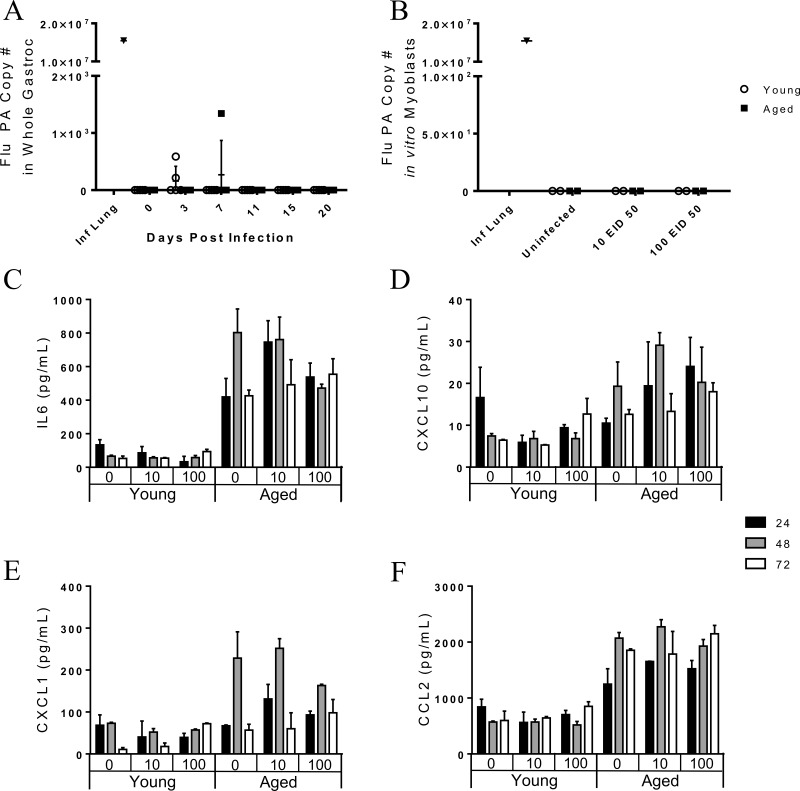
The pathogenesis of influenza-induced myopathies is likely not direct infection of skeletal muscle in vivo as viral copies are not seen in the gastrocnemius muscle Young and aged C57BL/6 mice were intranasally infected with 500 EID_50_ of PR8 influenza. (**A**) On day 0, 3, 7, 11, and 15 whole gastrocnemius was harvested and RNA was isolated. Total number of copies of influenza PA was determined via RT-qPCR with a positive control used (infected mice lung tissue). Uninfected young and aged murine myoblasts were incubated with 0, 10, or 100 EID_50_ PR8 influenza for one hour and then were cultured in growth media. Myoblast supernatant was analyzed for chemokine/cytokines via multiplex assay. Detectable cytokines were analyzed by 3-way ANOVA (age x infection condition x time point). While a significant age effect was observed (p<0.05), there was no effect of infection and no interaction of infection and time for myoblast secretion of IL6 (**C**), CXCL10 (**D**), CXCL1 (**E**), and CCL2 (**F**). At 96 hr post infection, total RNA from the myoblast culture was extracted and total number of copies of influenza PA was determined via RT-qPCR. No viral copies were present in in vitro myoblast cultures (**B**).

Since flu-induced weight loss was correlated with gastroc gene expression, we next examined a potential mechanism; direct infection of the skeletal muscle *in vivo*. RNA harvested from the gastroc was probed for the flu PA similar to as performed on whole lung tissue. Though rare case studies have identified virus particles in muscle biopsies of influenza infected humans with myalgia, this highly unusual finding may be limited to critically ill and preterminal cases [12, 21-23]. Indeed, flu PA was not detectable in the majority of gastroc tissue and the few samples that had detectable levels of flu PA had only negligible levels (Fig 7A). Since there was an upregulation of muscle degradation genes in all mice by day 7 post infection, the mechanism of direct infection occurring during a natural infection is not supported by these results.

### In vitro myoblasts do not harbor active influenza infection

To further investigate the possibility that direct infection of skeletal muscle could occur, we harvested leg skeletal muscle (gastroc, vastus medialis, vastus lateralis, soleus, and anterior tibialis) of uninfected young and aged mice. Myoblasts were harvested, grown, purified, and re-plated prior to infection. Myoblasts were incubated for 1 hour with 0 EID_50_, 10 EID_50_, or 100 EID_50_ PR8. Supernatants were collected at 24, 48, and 72 hours post infection and cytokine/chemokine concentration were determined via multiplex (Fig 7C, 7E, and 7F). IL-6 secretion was significantly greater from the aged myoblasts, (age effect: p<0.001), but was not affected by infection or infection over time (infection effect: p=0.600, infection*time interaction effect: p=0.583, Fig 7C). The same pattern was observed for CXCL10 (age: p<0.001, infection: p=0.609, infection*time: p =0.684, Fig 7D), CXCL1 (age: p<0.001, infection: p=0.865, infection*time: p =0.121, Fig 7E), and CCL2 (age: p<0.001, infection: p=0.921, infection*time: p =0.263, Fig 7F). Thus, cytokine secretion was affected by age, but not by flu infection. Myoblasts were harvested and RNA was extracted at 96 hours post infection to probe for flu PA copies as previously done. Though some studies [18-20] show that myoblasts can be infected by influenza *in vitro*, our results indicate that both young and aged myoblasts are not susceptible to a productive flu infection (Fig 7B).

## DISCUSSION

Studies addressing the pathophysiology of flu infection typically focus on respiratory and immune systems, while those seeking to understand aging-related declines in mobility performance emphasize muscle biology and relevant neural systems. Nevertheless, it has become clear that systems-based approaches are essential to both aging research and clinical care of the elderly since a failure to think more broadly fails to consider crosscutting biological themes and motifs in aging and also ignores crucial bidirectional signals between different systems and tissues. With these considerations in mind, we have investigated the potential clinical significance of flu-associated myalgia and myopathies in relation to flu-induced disability in the elderly. Indeed, we have shown that flu infection induces both functional decrements and upregulation of muscle inflammation and atrophy gene expression that is more pronounced with aging, indicating the impact of flu infection on muscle may directly predispose elderly for catastrophic disability and sarcopenia. Additionally, the functional alterations observed during flu infection may increase risk of falls and other musculoskeletal injuries. Thus, here we have identified flu infection as a previously unrecognized, but potentially targetable, inducer of muscle atrophy potentially leading to decreased resilience in the elderly.

Aged mice have more severe and prolonged weight loss, as well as increased lung viral titers and delayed viral clearance following sublethal flu infection [35]. Similarly, voluntary locomotor activity is decreased with flu infection, and this reduction is prolonged in aged mice. However, since it is possible that voluntary activity could be diminished due to general flu-induced malaise, we analyzed gait patterns to assess more specific flu-induced alterations in functional performance. We showed that aged mice initially had a wider fore limb stance compared to young mice, but this was decreased with flu. It is known that elderly generally increase stance width to increase stability [36, 37], though this relationship is not yet established in mice. Midline distance was also decreased with flu infection and this was more dramatic in the hind limbs of the aged mice. Narrower steps with flu infection likely lead to decreased balance, potentially leading to increased risk for injury, as narrower stride width is associated with increased fall risk in elderly humans [36]. Stride length variability and gait symmetry index also increased with flu infection and is more prominent in the aged mice. Indeed, gait variability is also a predisposing factor for falls in elderly [38, 39]. Additionally, we showed decrements in acceleration and deceleration parameters with flu infection in both young and aged mice. Declines in the rate of force development and power output are evident in the elderly, and more importantly, are strong predictors of functional status and falling risk [40, 41]. Thus, many flu-induced functional alterations could be particularly problematic for already at-risk elderly.

Since functional alterations were primarily in the hind limbs we examined the gastroc, a large mixed fiber type muscle, for flu induced alterations in gene expression. Importantly, we demonstrated that flu-induced functional decrements were associated with increased inflammatory and atrophy gene expression. Both young and aged gastroc had increased expression of *IL6* and *IL6RA* by 7 DPI, while *IL6RA* expression remained elevated at 11 DPI only in aged mice. Additionally, *TNF* expression was only increased in the aged mice. Though the relationship between IL-6 and muscle inflammation and regeneration is not completely clear, higher expression of IL-6 and TNF in elderly skeletal muscle is associated with decreased muscular strength [42]. Further, it has been demonstrated that elderly humans have elevated expression of inflammatory mediators, particularly IL-6 and TNFα, and dysregulated signaling responses that lead to an increased inflammatory milieu and impaired myogenesis [43]. This low grade inflammation with aging also impairs postprandial muscle protein synthesis [44]. In addition to the common inflammatory mediators in muscle, *CXCL10* expression was dramatically increased only in the aged mice. While CXCL10 is predominantly associated with a Th1 response, it has been recently identified in inflammatory myopathies [45, 46], and secretion of CXCL10 from human fetal skeletal muscle cells is induced by treatment with either interferon (IFN)-γ or TNFα [45]. The dramatic increase in *CXCL10* expression in the aged mice contributes to exaggerated and prolonged gastroc inflammation. Thus, our results agree with previous research utilizing chemical [47] or exercise injury [43] and suggests that flu induces muscle inflammation in the aged that is heightened and prolonged potentially leading to further muscle damage and diminished regeneration.

Indeed, lingering inflammation in the aged was accompanied by increased and prolonged expression of atrophy and protein degradation genes. At 7 DPI both young and aged mice gastroc had increased expression of *ATROGIN1* and *MuRF1*; however, at 11 DPI while young mice downregulated expression, aged mice atrophy gene expression remained elevated. Similarly, both *UBB* and *UBC* expression was significantly higher in aged mice at 11 DPI. Proteins targeted for degradation that lead to muscle atrophy by atrogin-1 include myogenic regulatory factor MyoD and eukaryotic translation initiation factor 3 subunit f (eIF3-f), while MuRF1 preferentially targets myosin heavy chains and other myofibrillar proteins, though these targets are likely not exclusive to either ligase [30]. Further, in many instances, UBC acts in concert with atrogin1 and MuRF-1 through FOXO dependent pathways [48] suggesting that the increased *FOXO1* expression may tie together these proteolytic pathways in our flu induced muscle atrophy model. Moreover, increased *UBC* expression has been one of the most prominent mRNA increases observed in multiple muscle wasting disorders [49], so it is not surprising that *UBC* expression was the highest correlated gene with weight loss. Indeed, multiple atrophy and degradation genes were correlated with flu-induced weight loss, suggesting flu-induced weight loss is at least partly due to muscle degradation and atrophy. Interestingly, *IGF1* was negatively correlated with weight loss in young mice only. Also, in the step-wise multiple regression model, the addition of *IGF1* accounted for greater explanation of variability in weight loss in the young mice; however, this was not observed in the aged mice, suggesting anabolic signals are not strong contributors to percent weight loss and recovery in aged mice. Indeed, this lack of relationship observed with *IGF1* expression in the aged mice is likely attributable to anabolic resistance, a more recent concept described as the diminished response in aged muscle to many anabolic stimuli including branched chain amino acids and exercise [50, 51].

The observed suppression of positive regulators of muscle mass further tips the protein synthesis and protein degradation balance; and these were greater suppressed and prolonged in the aged mice. *IGF1* and *MEF2C* suppression was prolonged to 11 DPI in the aged mice, and peak suppression of *PAX7, MYOD1,* and *MYOG* was greater in aged mice compared to young mice. Certainly, IGF1 has been of particular interest in aging research over the years, and low circulating levels of IGF1, particularly in combination with elevated IL-6, have been associated with decreased muscle strength and increased prevalence of sarcopenia [52]. Thus flu induces these unfavorable responses in muscle tissue itself, predisposing the aged muscle to sarcopenic conditions. Surprisingly, no upregulation of positive regulators was evident during our time course, suggesting muscle mass may not be recovered.

Together our results suggest that flu induces functional decrements, as well as muscle inflammation, proteolysis, and atrophy, and that these changes are augmented and prolonged with aging. The pathogenesis behind this effect still remains unknown. Despite some studies suggesting direct infection of muscle cells [18-20], we showed no viral copies in gastroc muscle *in vivo* throughout the infection. Additionally, myoblasts were not susceptible to a productive infection *in vitro*. Desdouits et al. [19] reported that human myoblasts were less susceptible to flu infection than myotubes and response was variable among donors and flu strain, however many reports regarding productivity of infection in myoblasts and myotubes are conflicting [18-20, 53]. Further, it is important to note that mature muscle fibers are much different than myoblasts and myotubes, where these immature muscle cells exist only transiently. Satellite cells are only present in great quantities in early postnatal development and decrease dramatically in adulthood, accounting for 30-35% then 2-7%, respectively, of sublaminal nuclei on myofibers [54]. Supporting this, Nevalainen et al. [53] showed that mature muscle fibers do not produce viral progeny, though a non-permissive infection occurs. Indeed, *in vivo* studies have shown that a non-permissive infection may occur in the skeletal muscle [25], though this seems more likely when intramuscular flu infection models are used [24, 26]. Since we performed intranasal infections, similar to the natural route of infection in humans, it is unlikely this would occur; however, it is still possible that this non-permissive infection leads to viral copies below our detectible limit and direct viral infection, or perhaps the presence of viral particles, may contribute to muscle degradation. Collectively, the lack of *in vivo* evidence to suggest that mature muscle fibers are susceptible to infection and that this would actually occur during a natural flu infection leads us to conclude further research is necessary to determine the mechanism behind flu-induced muscle inflammation and degradation.

In summary, this manuscript is the first to identify in a controlled experiment setting flu-induced muscle inflammation and atrophy as well as functional impairment. Further, these effects are prolonged with aging, providing a molecular link to flu infection and disability in the elderly, together with some initial insights into the mechanism which may underlie aging-related declines in resilience. We have demonstrated that key inflammatory signals, and key ubiquitin proteasome components, both atrogin1 and MuRF1, as well as ubiquitin B and ubiquitin C, are upregulated. As it is known that muscle repair is diminished with aging, it is likely these muscle losses are not easily recoverable. Thus, future research may be able to target these pathways to prevent flu-induced atrophy and potential loss of quality of life in the elderly.

## METHODS

### Mice

Young (2.5-4 month old) C57BL/6 male mice were obtained from Charles River Laboratories and aged (19-22 month old) C57BL/6 male mice were obtained through the National Institute on Aging rodent colony. All mice were housed in a climate controlled environment with 12:12 light:dark cycle and fed standard rodent chow and water ad libitum. All procedures were approved by the University of Connecticut Medical School IACUC (protocol 100705) and carried out in accordance with these regulations. All mice underwent gross pathological examination at time of sacrifice and animals with obvious pathology were excluded from the study.

### Viral infection

Mice were anesthetized with isoflurane and intranasally inoculated with 50μl of 500 EID_50_ of influenza virus A/PR/8/34 (PR8). Mice were weighed daily to monitor infection progression. At time points indicated, whole lung tissue was homogenized and RNA was isolated via RNeasy Mini Kit (Qiagen Inc., Valencia, CA). RNA was reverse transcribed with iScript cDNA synthesis Kit (Bio-Rad Laboratories, Inc., Hercules, CA) and flu viral copies were detected via reverse transcription quantitative PCR of flu acid polymerase (PA).

### Voluntary locomotor activity

Spontaneous voluntary locomotor activity was measured via open field test at time points indicated. All tests were performed between 6-8am to control for diurnal variations. Following acclimation to the dim-lit testing room (at least 1 hour), mice were placed in the center of the photobeam activity system-open field (PAS-OF, 16”x16”x15” acrylic animal enclosure, San Diego Instruments, San Diego, CA) and their activity was recorded for 20 minutes. The first 5 minutes was excluded as this is generally considered to be exploratory behavior rather than general voluntary locomotor activity. The number of beam breaks per minute during the last 15 minutes was then used to assess voluntary locomotor activity.

### Gait analysis

Gait analysis was performed using the DigiGait instrument (Mouse Specifics, Inc, Quincy, MA) and software (DigiGait Imager 4.0.0 and DigiGait Analysis 11.5, Mouse Specifics, Inc). The DigiGait instrument consists of a clear treadmill with a high-speed camera mounted underneath that collects images at 147 frames per second for high resolution of postural temporal gait parameters. Mice run within a 2” wide acrylic running chamber at set speeds. The ventral plane videos are analyzed with the DigiGait software which identifies portions of the paw that are in contact with the treadmill belt to produce both postural and kinematic gait parameters. Mice were introduced to the DigiGait system at a low speed (10cm/sec) briefly (30 seconds) prior to the initial testing. Mice were allowed to acclimate to the dim-lit room for 1 hour prior to each testing period and all tests were performed between 6-8am. Mice ran at the testing speed (16cm/sec) until approximately 5 seconds of consecutive walking was recorded and this video segment was analyzed via DigiGait software.

### Gastrocnemius Reverse-Transcription quantitative PCR (RT-qPCR)

At the time points indicated for gastrocnemius gene expression mice were fasted with the exception of water for 4-6 hours prior to sacrifice to minimize potential confounding results due to postprandial muscle protein synthesis. The gastrocnemius muscle was dissected and placed in RNAlater (Qiagen Inc.) overnight at 4°C. RNAlater was removed and gastrocnemius was frozen at 80°C until RNA extraction. The muscle was homogenized and RNA was extracted via RNeasy Fibrous Tissue Mini Kit (Qiagen Inc.). RNA quantity and quality was assessed with Nanodrop 2000c (Thermo Scientific, Waltham, MA) and was reverse transcribed via iScript Advanced cDNA synthesis Kit (Bio-Rad Laboratories, Inc.). RT-qPCR was performed using custom designed PCR plates with predesigned commercially available primers (Bio-Rad Laboratories, Inc.). Gene expression was calculated via a modified Pflaffl method utilizing multiple reference genes (RPS18 and TBP, which showed the least variability between conditions and thus suitable reference genes) and normalized to gene expression of young mice at Day 0 prior to infection to give comparable fold changes.

### In vitro myoblast culture and infection

Harvested leg skeletal muscle (gastroc, vastus medialis, vastus lateralis, soleus, anterior tibialis) from uninfected young and aged mice was incubated in collagenase type IIA and dissociated in growth media (Dulbecco modified Eagle's medium (D-MEM) supplemented with 20% fetal bovine serum (FBS), 1% penicillin/streptomycin, 5ng/mL basic fibroblast growth factor (bFGF)) and grown on extracellular matrix-coated plates (extracellular matrix, Sigma-Aldrich Corp., St. Louis, MO). When myoblasts reached 50-60% confluency, they were purified via plating on an uncoated Petri dish to remove any adherent fibroblasts. Myoblasts were then seeded at 150,000 cells per well in coated 6-well plates and allowed to grow to 50-60% confluency prior to infection. For infections, myoblasts were washed twice with phosphate buffered saline (PBS) and incubated for one hour with 0, 10, or 100 EID_50_ PR8 in D-MEM supplemented with 1% penicillin/strepto-mycin. Then myoblasts were washed twice with PBS and cultured with growth media for the remaining days. Culture media at time points indicated was analyzed for cytokine/chemokine concentrations via multiplex (25Plex Magnetic Bead Panel, EMD Millipore, Billerica, MA). Most cytokine/chemokines were below detectable limits (GCSF, GMCSF, IFNγ, IL10, IL12p40, IL12p70, IL13, IL15, IL17, IL1β, IL1α, IL2, IL4, IL5, IL7, IL9, MIP1β, MIP1α, MIP2, RANTES, TNFα). Total RNA was extracted via TRIzol (Ambion, Life Technologies, Grand Island, NY) according to manufacturer's recommendation. RNA was reverse transcribed and flu viral copies were detected via real-time quantitative PCR of flu PA.

### Statistical analysis

Weight loss, viral titers, functional performance, and log-transformed gastroc gene expression results were analyzed via 2-way ANOVA (age x time point) with Bonferroni post hoc corrections when indicated with significance set a p<0.05. Genes were considered differentially expressed if fold changes were ≥ 2 and p<0.05. In vitro myoblast supernatant and RNA were analyzed via 3-way ANOVA (age x infection condition x time point) and 2-way ANOVA (age x infection condition), respectively, with Bonferroni post hoc corrections when indicated with significance set at p≤0.05. Univariate linear regression was used to compare the relationship between gastroc gene expression and percentage weight loss independently for each variable that showed time effects with significance set at p<0.05. Step-wise multiple regression analysis was used to determine if multiple variables could better predict percent weight loss.
